# COMPASS: A computational model to predict changes in MMSE scores 24-months after initial assessment of Alzheimer’s disease

**DOI:** 10.1038/srep34567

**Published:** 2016-10-05

**Authors:** Fan Zhu, Bharat Panwar, Hiroko H. Dodge, Hongdong Li, Benjamin M. Hampstead, Roger L. Albin, Henry L. Paulson, Yuanfang Guan

**Affiliations:** 1 Department of Computational Medicine and Bioinformatics, University of Michigan, USA; 2 Department of Neurology and Michigan Alzheimer’s Disease Center, University of Michigan, USA; 3 Department of Neurology and Layton Aging and Alzheimer’s Disease Center, Oregon Health & Science University, USA; 4 Department of Psychiatry, University of Michigan, USA; 5 Mental Health Service, VA Ann Arbor Healthcare System, USA; 6 Neurology Service & Geriatric Research Education and Clinical Centers, VA Ann Arbor Healthcare System, USA; 7 University of Michigan Udall Center, USA; 8 Departments of Internal Medicine and Human Genetics, and School of Public Health, University of Michigan, USA; 9 Departments of Internal Medicine and of Electrical Engineering and Computer Science, University of Michigan, USA

## Abstract

We present COMPASS, a COmputational Model to Predict the development of Alzheimer’s diSease Spectrum, to model Alzheimer’s disease (AD) progression. This was the best-performing method in recent crowdsourcing benchmark study, DREAM Alzheimer’s Disease Big Data challenge to predict changes in Mini-Mental State Examination (MMSE) scores over 24-months using standardized data. In the present study, we conducted three additional analyses beyond the DREAM challenge question to improve the clinical contribution of our approach, including: (1) adding pre-validated baseline cognitive composite scores of ADNI-MEM and ADNI-EF, (2) identifying subjects with significant declines in MMSE scores, and (3) incorporating SNPs of top 10 genes connected to APOE identified from functional-relationship network. For (1) above, we significantly improved predictive accuracy, especially for the Mild Cognitive Impairment (MCI) group. For (2), we achieved an area under ROC of 0.814 in predicting significant MMSE decline: our model has 100% precision at 5% recall, and 91% accuracy at 10% recall. For (3), “genetic only” model has Pearson’s correlation of 0.15 to predict progression in the MCI group. Even though addition of this limited genetic model to COMPASS did not improve prediction of progression of MCI group, the predictive ability of SNP information extended beyond well-known APOE allele.

Alzheimer’s disease (AD) is a chronic neurodegenerative disorder accounting for approximately 60% of dementia cases^1^. Approximately 21 million people worldwide have AD^2^, with approximately 2.4 million people affected in the United States^3^,^4^,^5^. AD is the 6^th^ leading cause of death in the United States. The majority (96%) of individuals with AD are older than 65 years with approximately exponential increases in incidence and prevalence accompanying aging^4^. About 11% of people at age 65 are affected by AD, and prevalence increases to 30% to 50% among people age 85 or older^6^.

It is now widely accepted that AD is a multi-decade process with a long asymptomatic phase^7^. The spectrum of AD includes a period preceding overt dementia when affected persons experience identifiable cognitive deficits without substantial functional impairments; the phase of Mild Cognitive Impairment (MCI)^8^. It is also widely believed that effective preventive treatment will need to be provided at early phases of the AD spectrum^9^. Disease progression is slow during the earliest or preclinical stages of AD^10^. Early identification of those at risk for developing AD and accurate prediction of cognitive trajectories in individuals with MCI are particularly important as preventive therapies are sought for AD. The MCI phase is of particular interest because individuals in this phase are easier to identify than presymptomatic individuals and consequently a key population for natural history and intervention studies.

Several attempts have been made to use clinical and biomarker data to predict progression of MCI to AD. Kovacevic *et al*. developed an automated method that measured segmental Medial temporal lobe (MTL) volumes to predict clinical decline in MCI patients^11^. Davatzikos *et al*. predicted MCI to AD conversion by combining information from MRI, CSF biomarkers, and pattern classification^12^. Zhang *et al*. predicted progression of MCI patients using longitudinal and multimodal biomarkers^13^. While the hazard levels of clinical parameters were evaluated in previous studies, accurate models designed to identify those at risk of developing AD are not available. Prior prediction methods also used different datasets, making it difficult to compare predictions. Use of multimodal biomarkers may not be feasible for clinical practice and is inconvenient for clinical research.

It was on this background of prior studies that the Dialogue for Reverse Engineering And Methods (DREAM) Alzheimer’s Disease Big Data challenge^14^ was organized to identify efficient and comparable methods using a ‘*wisdom of the crowd*’ approach. In this Challenge competition participants were asked to come up with an algorithm which predicts the decline in Mini-Mental State Examination (MMSE) scores over 24 months using a pre-specified data set. The MMSE is a 30-point questionnaire used widely in clinical and research settings to measure cognitive status in dementias^15^,^16^. Significant community-wide efforts^17^ are devoted to developing methods to predict significant declines in MMSE scores^18^,^19^,^20^. As part of the DREAM Challenge, we developed COmputational Model to Predict the development of Alzheimer’s diSease Spectrum (COMPASS) to predict significant declines in MMSE score (delta MMSE) 24 months after initial assessments. In this blinded, crowdsourcing competition, our method outperformed other participating methods^21^. The short computational time of this method, requiring only a few seconds to establish the model and make predictions, makes it good candidate for clinical research use and for potential clinical applications.

In an effort to improve COMPASS and to explore personalized predictions, we further included cognitive composite scores as predictors of MMSE declines, and evaluated the feasibility of using genetic information other than APOE to predict delta MMSE in the MCI population.

## Materials and Methods

### Data acquisition

Data used in the preparation of this article were obtained from the Alzheimer’s Disease Neuroimaging Initiative (ADNI) database (adni.loni.usc.edu). ADNI was launched in 2003 as a public-private partnership, led by Principal Investigator Michael W. Weiner, MD. The primary goal of ADNI is to test whether serial magnetic resonance imaging (MRI), positron emission tomography (PET), other biological markers, and clinical and neuropsychological assessment can be combined to measure the progression of mild cognitive impairment (MCI) and early Alzheimer’s disease (AD).

For the current study, we used ADNI-1 data downloaded on February 10^th^, 2015. ADNI-1 general eligibility criteria are described at ADNI website. Normal subjects had MMSE scores between 24 and 30 (inclusive), Clinical Dementia Ratings (CDR) of 0, and did not meet criteria for MCI or AD. MCI subjects had MMSE scores between 24 and 30 (inclusive), a memory complaint, objective memory loss measured by education adjusted scores on the Wechsler Memory Scale Logical Memory II test^22^, CDRs of 0.5^23^, absence of significant levels of impairment in other cognitive domains, essentially preserved activities of daily living, and absence of dementia. Mild AD subjects had MMSE scores between 20–26 (inclusive), CDRs of 0.5 or 1.0, and met NINCDS/ADRDA criteria for probable AD^24^.

We downloaded the following information: age, gender, education, Apolipoprotein E (APOE) genotype, neuropsychological tests and validated composite scores based on the results of neuropsychological test results (ADNI-MEM for memory and ADNI-EF for executive functions) at baseline, and the MMSE scores at baseline and at the 24-months follow up assessments. ADNI-MEM and ADNI-EF are composite measures of memory and executive function, respectively, derived from the ADNI neuropsychological battery^25^,^26^. Since we were also interested in exploring predictive genetic information, we limited data to individuals for whom Single Nucleotide Polymorphism (SNP) data were available. Of the 489 individuals in this dataset, 152 of them are cognitively normal (CN), 230 of them are MCI, and 107 of them are AD patients at baseline (Table 1).

### Data pre-processing

We reformed APOE genotype as two features: the number of APOE2 (rs429358, *the number of APOE2 is 2 minus the frequency of rs429358) and the number of APOE4 (rs7412). For genetic features, the dosage of each SNP is calculated from their genotypes as 2 × *AA* + 1 × *AB* + 0 × *BB*.

Individuals were split into three groups (CN, MCI and AD) based on their diagnosis. Within each group, all features are then scaled to [0, 1], including genetic features.

### Genetic Data Retrieval

To explore personalized predictions, we made use of patients’ SNP data in our genetic model. Besides the well-known APOE bio-marker^27^, we are also interested in exploring how other genetic information could contribute to our method. To select the SNPs features, we built a brain-specific functional relationship network by integrating multi-species genomic data using Bayesian network^28^,^29^,^30^,^31^. In this network, each node represents a gene and an edge indicates the probability that two genes participate in the same biological process or a common pathway. We identified 10 genes closely functionally related to APOE, including *PRNP* (prion protein), *APOA1* (apolipoprotein A-I), *APP* (amyloid beta precursor protein), *GRID2* (glutamate receptor, ionotropic, delta 2), *MT1H* (metallothionein 1H), *PSAP* (prosaposin), *MT1E* (metallothionein 1E), *GJA1* (gap junction protein, alpha 1, 43 kDa), *EDNRB* (endothelin receptor type B) and *CBS* (cystathionine-beta-synthase). We then queried these genes using NCBI dbSNP^32^ and obtained 13,347 associated SNPs (Supplementary Dataset S1). As we explored the contributions of other genetic information to our prediction method, APOE genotype was excluded as it is already a well-known bio-marker.

### Implementation

Within each group, we used multiple learning methods to model delta MMSE. Our method is implemented using Support Vector Machine (SVM) light^33^ with linear and Radial Basis Function (RBF) kernel functions. SVM is a maximal margin classifier that maximize the distance of the nearest correctly classified examples to optimize classification performance. SVM aims at minimizing the cost function:


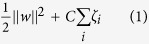


where w defines the plane that separates the positive and negative examples, ζ represents the degree of misclassification for each sample, C is a constant which is empirically optimized.

SVM can locate hidden disease development patterns through only a few samples in a training dataset and SVM may automatically identify disease development patterns whose combination maximally separates individuals developing into AD from controls.

Linear kernel is widely used and the simplest kernel function, defined as:


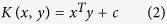


where *x*^*T*^ *y* is the inner product between samples x and y, c is an optional constant parameter.

RBF kernel is a popular kernel function in learning algorithms. RBF kernel calculates the distance between x and y by:


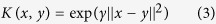


where ||*x* − *y*|| is the Euclidean distance between the feature vectors of training samples. *γ* is a free parameter determined using cross-validation.

Our method to predict individuals meeting the delta MMSE criterion (discussed later) in the all three groups (CN, MCI, and AD), we tried SVM with both linear kernel and RBF kernel. The trade-off parameter *c* and gamma (*γ*) were optimized using computational cross-validation. Age, gender, education, baseline MMSE evaluation, and APOE genotype were used as the input features for SVM. We evaluated predictive accuracy with and without baseline ADNI-MEM and ADNI-EF.

### Comparison Methods

The comparison methods, *i.e.* Gaussian process regression (noise = 1, γ = 2)^34^,^35^, linear regression, RBF Network, support vector machine for regression (or SMOreg, c = 1.0, E = 1, 2, or 3), decision tree, are implemented using WEKA^36^. Gaussian process regression, or Kriging, is a regression method based on Gaussian process governed by covariance function. RBF network, or radial basis function network, is an artificial neural network that uses RBF functions as activation functions. SMOreg is the support vector machine for regression implemented by WEKA, where the polynomial kernel is used and E parameter of indicates its exponent value.

As described above, individuals were separated into three groups based on their baseline diagnoses of CN MCI and AD. Separate models were trained for each group (Supplementary Info S2).

### Significant Decline in MMSE

Predicting declines in MMSE score as continuous variables from baseline to 24 months using ADNI data was a DREAM Challenge Question^14^. In the current study, we further identified those with a significant decline in MMSE. We defined significant delta MMSE as a decline of 3 points, as indicated by prior research^18^,^37^. Predictive accuracy was evaluated using two criteria: the magnitudes of Pearson and Spearman’s correlation coefficients. We used both because both are widely used measures with somewhat different but relevant properties. The Pearson correlation coefficient is the standard method for parametric data while Spearman’s correlation coefficient is the preferred method for non-parametric data and more robust with respect to the effects of outliers. The correlation coefficients between the observed changes in observed delta MMSE scores and predicted delta MMSE were calculated. Predictive ability of our model in identifying those with significant MMSE decline was estimated by Receiver Operating Characteristics Area Under Curve (ROC AUC).

## Results

We developed customized kernel-based models to predict delta MMSE. Because of differences between AD, Mild Cognitive Impairment (MCI) and Cognitive Normal (CN) groups, we developed separate models to predict delta MMSE for each group. We also evaluated accuracy when combining individuals from all three groups.

### Clinical Data Summary

489 individuals from ADNI-1 study were used in our analysis, including 152 CN, 230 MCI and 107 AD. As shown in Table 1, average MMSE scores were 27.0 at baseline and 25.2 at the 24-month assessments. The average age was 75.4 years, and average year of education was 15.7 where 59.7% were male. There were 2 (out of 152) significantly declining individuals in the CN group, 77 (out of 230) in the MCI group, and 60 (out of 107) in the AD group, for a total number of 139 (out of 489) significantly declining individuals.

### MMSE changes from baseline to 24-months

**Figure 1:**
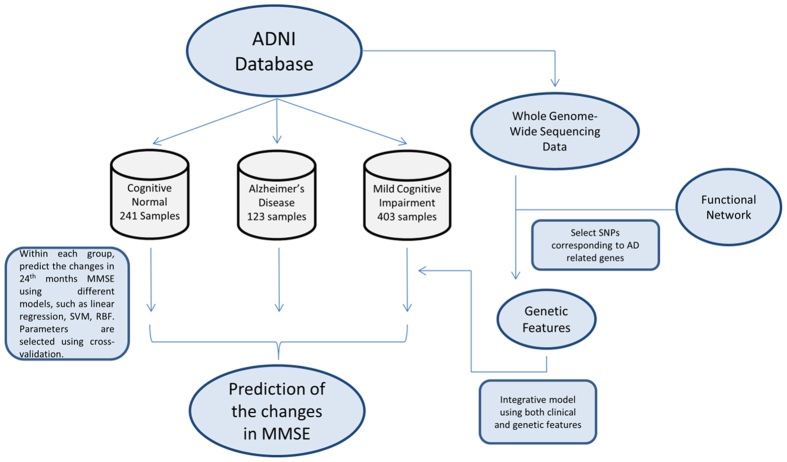
Workflow of our method. This figure shows the overview of our method. Individuals are separated into three groups based on their diagnosis and separate models are applied to each group. Additionally, to explore the predictive power of new genetic information, genetic features are extracted from SNP data as the input of the integrative model to predict the development of MMSE for MCI individuals.

Figure 1 is a workflow figure indicating how we established the COMPASS model to predict delta MMSE from baseline to the 24 month assessment. We divided individuals into three diagnosis groups and different models were applied to each group.

#### (a) COMPASS predictive accuracy without ADNI-MEM and ADNI-EF

We performed computational cross-validation to evaluate the predictive accuracy of COMPASS. For each diagnosis group and parameter, correlation coefficients between predicted and observed changes in MMSE scores are calculated by repeating ten-fold cross-validation 10 times. COMPASS is implemented based on SVM light^33^. The comparison methods are evaluated using WEKA, where 10 fold cross-validation is also repeated 10 times.

**Figure 2:**
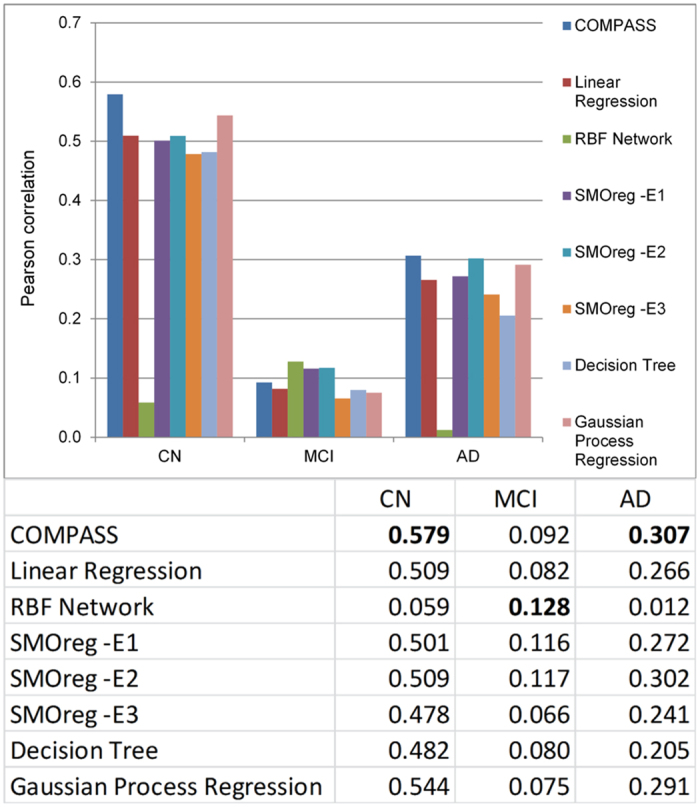
The performance of COMPASS vs. other methods (without ADNI-MEM and ADNI-EF). The performance of all methods is evaluated by repeating 10-fold cross-validation 10 times. Gaussian process regression, or Kriging, is a regression method based on Gaussian process governed by covariance function. RBF network, or radial basis function network, is an artificial neural network that uses RBF functions as activation functions. SMOreg is the support vector machine for regression implemented by WEKA, where the polynomial kernel is used and parameter E indicates its exponent value.

Figure 2 shows the performance comparisons between our method and other popular machine learning methods including linear regression, RBF network, support vector machine, decision tree, and Gaussian process regression.

Using COMPASS, the Pearson (Spearman’s) correlation between predicted and observed delta MMSE was 0.579 (0.469) for the CN group and 0.306 (0.30) for the AD group. MCI group predictions based on SVM provided the lowest correlation with a correlation of 0.092 and 0.109 for Pearson correlation and Spearman’s correlation, respectively. The WEKA based RBF Network algorithm performed relatively better for MCI group and provided 0.128 Pearson correlations. We tried both SVM linear and RBF kernel but linear kernel performed better for all three groups (CN, MCI, and AD).

**Figure 3:**
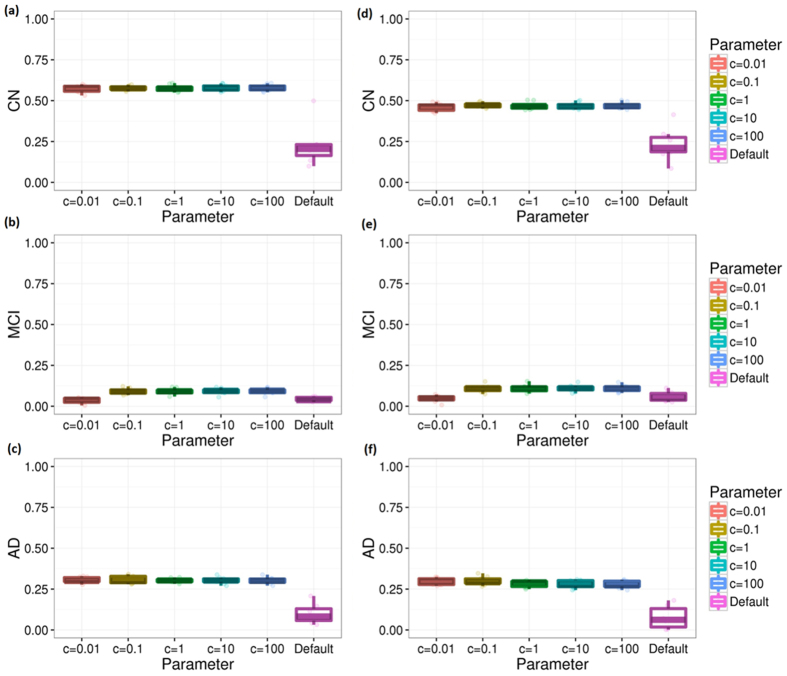
Cross validations for different diagnosis groups without ADNI-MEM and ADNI-EF. This figure shows the performance of COMPASS method measured by repeating 10-fold computational cross validation 10 times. Subfigures (**a**–**c**) are the results measured by Pearson correlation coefficient between the observed and predicted MMSE scores under different parameters. Subfigures (**d**–**f**) are the results measured by Spearman’s correlation coefficient.

Parameter optimization performed in COMPASS might improve predictive accuracy. Accordingly, we performed computational cross-validation to select model parameters. Figure 3 shows the performance improvement from our parameter adjustment for the different groups. As shown in Fig. 3, the *c* parameter is optimized to 100 for the all groups. (The results shown in Fig. 2 were obtained using the optimal parameters selected).

#### (b) Predicting accuracy of COMPASS with ADNI-MEM and ADNI-EF

Two additional baseline values were obtained from ADNI-1 data: composite scores of memory (ADNI-MEM)^20^,^38^ and executive function (ADNI-EF)^25^,^26^. These two scores are psychometrically optimized scores derived from ADNI tests and externally validated^26^,^39^.

The feasibility of using baseline ADNI-MEM and ADNI-EF values to predict delta MMSE over 24-months was evaluated. Although ADNI-MEM and ADNI-EF are available at both first screening and 24-month follow up from the ADNI database, we only used the first assessment information.

**Figure 4:**
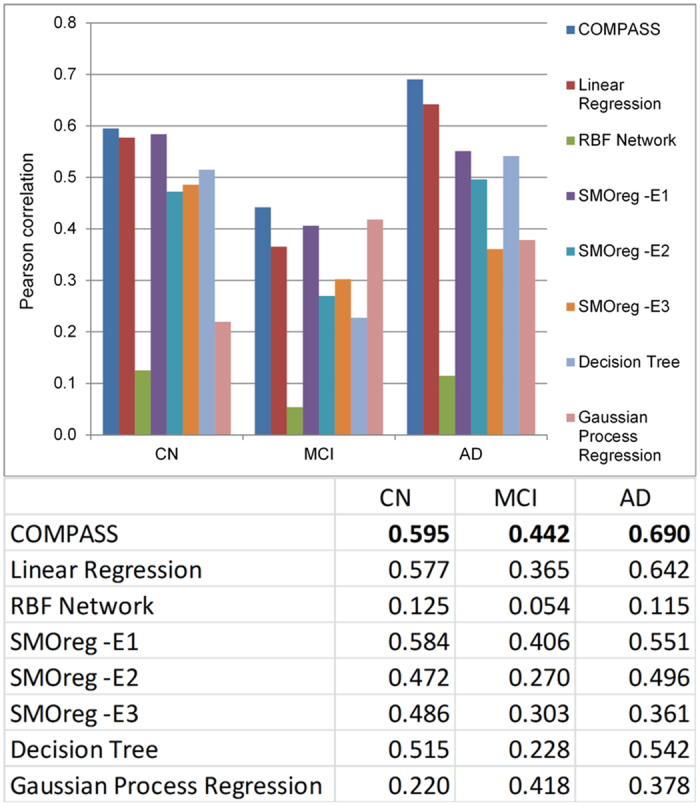
The performance of COMPASS vs. other methods (with ADNI-MEM and ADNI-EF). This figure shows the performance comparison of our method and other popular machine learning methods when the two ADNI cognitive composite scores are included. The performance of all methods is evaluated by repeating 10-fold cross-validation 10 times. The methods used here are the same as in Fig. 2.

Figure 4 shows the performance comparison between our method and other popular machine learning methods. For the CN group, the improvement brought by ADNI-MEM and ADNI-EF is minor and SVM with linear kernel has a Pearson’s correlation around 0.595 between predicted and observed changes in MMSE. For the MCI group, introducing ADNI-MEM and ADNI-EF significantly boosted predictive accuracy. The Pearson’s correlation coefficient between observed and predicted changes in MMSE is improved from 0.128 to 0.442. Among all the compared methods, Gaussian process regression (GPR) also delivers results with accuracy similar to our method. For the AD group, the performance improvement by adding the two composite scores was also dramatic; the Pearson’s correlation coefficient almost doubled from 0.306 to 0.690.

**Figure 5:**
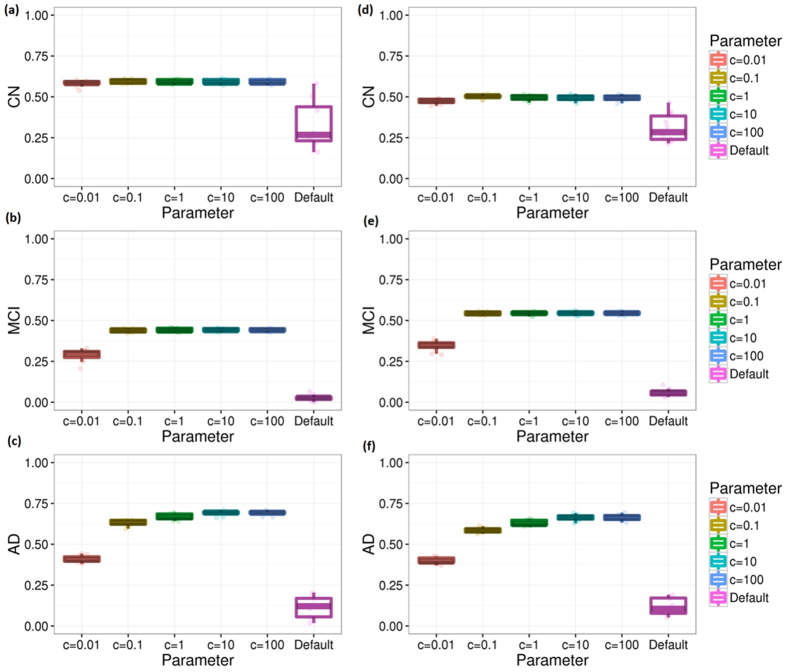
Cross validations for different diagnosis groups with ADNI-MEM and ADNI-EF. This figure shows the performance of our method measured by repeating 10-fold computational cross validation 10 times. Subfigures (**a**–**c**) are the results measured by Pearson correlation coefficient between the observed and predicted MMSE scores under different parameters. Subfigures (**d**–**f**) are the results measured by Spearman’s correlation coefficient.

Figure 5 shows how parameter optimization improves the accuracy. The optimal c parameters for CN, MCI, and AD group are 0.1, 10, and 100, respectively. The performance significantly improved with this parameter optimization.

#### (c) Predictions across all three groups

**Figure 6:**
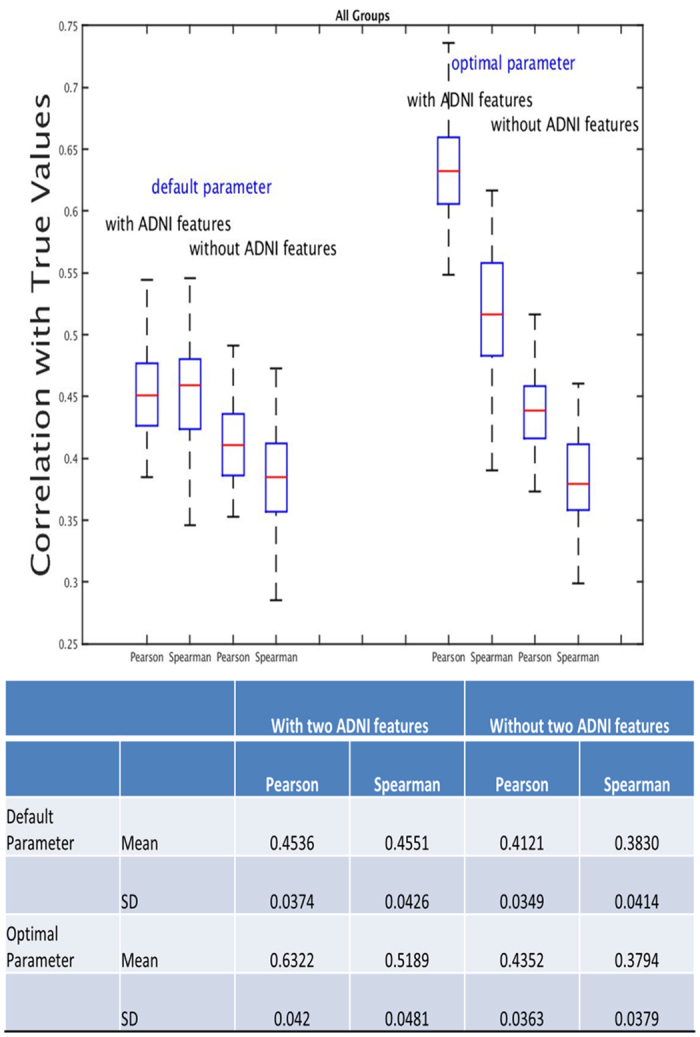
Cross validations across all three groups. This figure shows the cross validation performance of COMPASS across all three groups; ten-fold cross validation was repeated 100 times.

Figure 6 shows the overall performance for all individuals. Without two ADNI cognitive composite scores, the overall correlation is around 0.40 for Pearson and Spearman’s correlation coefficient. With ADNI-MEM and ADNI-EF, the accuracy is improved to approximately 0.45 for Pearson and Spearman’s correlation coefficients under default parameter. With parameter optimization, the predicting accuracy is further improved to 0.65/0.52 for Pearson/Spearman’s correlation.

### Predicting a large decline in MMSE within 24-months as measured by ROC and precision recall curves

MCI subjects vary widely in their rate of cognitive decline^40^, with some remaining as MCI, others transitioning to AD, and others reverting to normal. We measured how well our model could identify MCI individuals experiencing a clinically meaningful decline, defined as a MMSE decline of three or more points (77 individuals out of 230, about one third of the top decliners)^41^,^42^.

**Figure 7:**
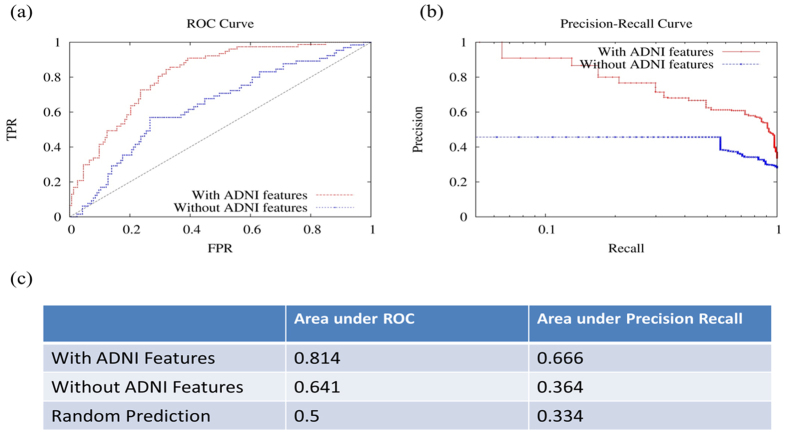
Prediction of MMSE decline of 3 points or more over 24-months in ADNI. This figure shows the ROC and precision recall curves for models with and without ADNI composite scores to predict those with MMSE declines of 3 or more points at 24-months from baseline.

Figure 7 shows the receiver operating characteristic (ROC) and precision recall curves. COMPASS delivered results with an area under ROC (AUROC) of 0.814 and an area under precision recall curve (AUPRC) as high as 0.666, compared with the 0.5 AUROC and 0.33 AUPRC for random prediction. The false discovery rate (FDR) is 0%, 9%, 20% at the 5%, 10%, 20% true positive rates (TPR or recall), respectively. In other words, the top 5% of significantly declining individuals predicted using our method is 100% correct; and our method maintains 80% accuracy for its top 20% predictions.

### Genetic model for MCI group

We introduced a genetic model to explore how genetic information could be used to predict delta MMSE in the MCI group. To select the genetic features for the integrative model, we queried genes that are closely related to APOE based on our gene co-functionality network^28^,^29^,^30^ and found the corresponding SNPs for the 10 most related genes (see Methods: Genetic data retrieving).

**Figure 8:**
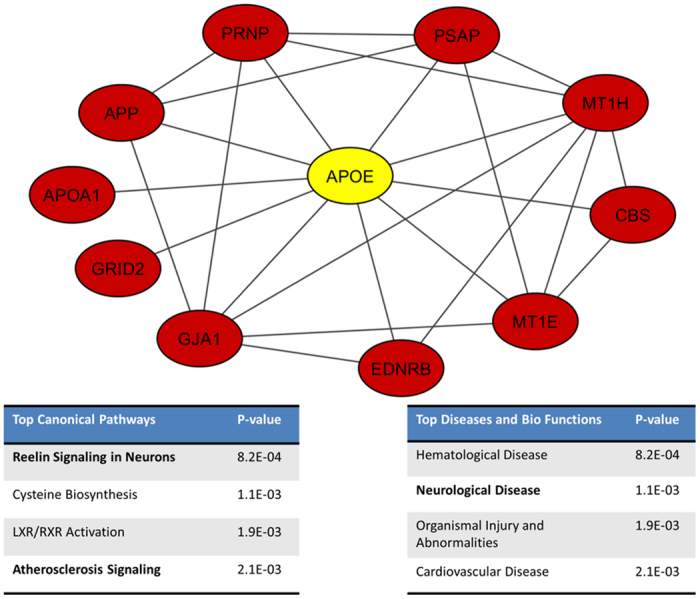
10 top connected genes of APOE. We used our co-functionality network to identify 10 neighbors of APOE, and extract the SNPs that correspond to these genes. These SNPs are then used as the input feature of our genetic model. The two sub tables show the top canonical pathways and top disease and bio functions of the 11 genes generated using Ingenuity IPA. These eleven genes are enriched to AD related pathways (Reelin Signaling in Neurons and Atherosclerosis Signaling) as well as neurological disease.

As APOE is a well-known genetic risk factor of AD, we employed it as a seed gene but excluded it from the genetic model. We built a brain-specific functional relationship network by integrating multi-species genomic data using a Bayesian network followed in our previous work^28^. This network indicates the gene pairs that are functionally related in brain context. We then identified the 10 most related genes with APOE from the network, and collected a list of SNPs corresponding to these 10 genes from the dbSNP database^32^. 13,347 SNPs were selected as the input features of our genetic model. Figure 8 shows the top 10 predicted neighbors of APOE. We performed pathway enrichment using commercial software, QIAGEN’S Ingenuity Pathway Analysis (IPA), for APOE and its 10 neighbors. The top enriched canonical pathways include Reelin Signaling in Neurons (p = 8.2E-04) and Atherosclerosis Signaling (p = 2.1E-03). Among the top disease families and biological functions enriched for these genes are neurological disease (p = 1.1E-03).

This genetic only model is able to deliver results with an accuracy of 0.15 (0.12) in terms of Pearson (Spearman’s) correlation coefficients, compared with the 0.13 (0.13) correlation for clinical only model (which includes APOE genotype) without ADNI composite memory and executive scores. This indicates that the 10 most connected neighbors of APOE (or other genes of interest in AD and other neurodegenerative diseases) could potentially be used as biomarkers, and that genome-wide association studies (GWAS) could provide good candidates to predict individuals’ delta MMSE. Adding the genetic model on top of our clinical model (with two ADNI cognitive composite scores) did not, however, further improve accuracy. We also applied the genetic only model to CN and AD groups. However, the results are close to random (correlation coefficient < 0.05).

### DREAM’s validation of methods on independent dataset

COMPASS was the best-performing approach in the DREAM^21^ Alzheimer’s Disease Big Data Challenge^43^. In the DREAM AD challenge, a blind assessment was made on data from the Religious Orders Study^44^ and the Memory and Aging Project^45^. That is, each competitor submitted their algorithm derived from ADNI data and DREAM competition organizers applied the models to the above cohort data (two cohort combined). Overall, 587 individuals (407 CN, 146 MCI, 34 AD) were used in the evaluation. MMSE was provided for the baseline, but not for the 24-month screening. Each participating group in the competition predicted MMSE scores at the 24-months using their best performing methods. Competition organizers checked the correlations between their data and the predicted scores provided by participating groups. 525 people across the world signed up for the challenge and 32 teams completed the challenge. Our proposed method was the best performing method in this challenge with correlation coefficients between predicted and observed MMSE scores of 0.382 and 0.433 for Pearson correlation and Spearman’s correlation, respectively^14^. This method was also the best performing method in a 100,000 bootstrapped assessment.

### Computational Time

COMPASS is computationally efficient. The clinical model of COMPASS takes less than one second to train and to make predictions on a common commercial computer (Dell PowerEdge R410). The running time of genetic and clinical combined model is less than one minute.

## Discussion

Predicting progression of cognitive impairment in individuals along the AD spectrum is important. While significant efforts have been devoted to the identification of hazard levels of clinical parameters, sophisticated models to accurately predict changes in cognitive status are not available. We used ADNI data to develop and evaluate a computational model, COMPASS, to predict significant changes in MMSE scores 24-months after initial assessment. Additional analyses beyond the question set by the DREAM AD challenge were conducted in the current study.

Without including the two ADNI cognitive composite scores, the Pearson (Spearman’s) correlations provided by COMPASS between observed and predicted values for changes in MMSE scores were 0.58 (0.47), 0.12 (0.13) and 0.31 (0.30) for CN, MCI and AD groups, respectively. Correlations were significantly improved by adding the cognitive composite scores at baseline, ADNI-MEM and ADNI-EF. The Pearson (Spearman’s) correlations were improved to 0.59 (0.50), 0.44 (0.54) and 0.69 (0.66) for CN, MCI and AD groups, respectively. The results indicate that these two ADNI measurements are highly predictive for MCI and AD groups, as their inclusion improves the performance significantly. On the other hand, the correlation is still in a moderate range, suggesting that using only clinical variables together with APOE information has a limitation in predicting changes in MMSE within 24 months. This could reflect the variability associated with MMSE test scores. For example, changes in MMSE by 1 point could be simply random variations associated with the test. When the outcome was decline in MMSE by 3 points, which is regarded as a clinically meaningful change, then COMPASS was able to deliver results with an area under ROC of detecting this outcome of 0.814. As indicated by precision recall curve, the top 5% of significantly declined individuals predicted using our method is 100% correct (*i.e.* 0% FDR), while the method remains 91% accurate for its top 10% recall (or TPR). The high accuracy in predicting individuals who most likely will experience significant decline in cognitive status makes COMPASS a strong candidate to assist in the identification of individuals progressing along the AD spectrum, and a potentially useful tool for enriching clinical trials.

The effectiveness of COMPASS was tested in the recent DREAM9 AD Big Data challenge, and found to be the best performing method (judged by both the Pearson and Spearman’s correlation between observed and predicted MMSE in an unseen dataset from Religious Orders Study and the Memory and Aging Project)^46^. The success of COMPASS in the DREAM challenge indicates that it is likely to work accurately in other datasets.

A first generation, limited “genetic only” model was developed to measure the predictive ability of SNP information beyond the well-known APOE allele effects. 13,347 SNP features are selected using our previously established co-functional networks. Our genetic model delivered results with an accuracy of 0.15 and 0.12 in terms of Pearson and Spearman’s correlation coefficients. However, adding this limited genetic model to our clinical model did not improve the accuracy. Possible reasons include: (a) APOE effects are relatively powerful and were properly excluded from this analysis; and (b) in a complex, polygenic phenotype, a few loci are not likely to be more informative than phenotypic markers like ADNI-MEM or ADNI-EF that reflect the cumulative effect of many genes/alleles as well as individual specific cognitive reserve^47^. It is worth investigating the interdependence between these genetic features and clinical features in future studies. More robust genetic models covering more pathogenic pathways may be good candidates to further improve COMPASS. Finally, it may be useful to include vascular disease factors in the model to enhance the prediction algorithm. Future studies will consider including these factors in the model.

COMPASS is efficient in terms of computational cost, which makes it highly scalable. It is ready to be released for the general research/clinical community. In its present form, COMPASS will allow clinical researchers to forecast significant changes in MMSE scores of potential research subjects in real time. This may be useful for selecting or stratifying subjects in clinical trials. With extensions, it may find a role in clinical practice.

## Additional Information

**How to cite this article**: Zhu, F. *et al*. COMPASS: A computational model to predict changes in MMSE scores 24-months after initial assessment of Alzheimer’s disease. *Sci. Rep.* **6**, 34567; doi: 10.1038/srep34567 (2016).

## Supplementary Material

Supplementary Dataset S1

Supplementary Info S2

## Figures and Tables

**Table 1:** Data Summary.

	CN	MCI	AD	All
#Samples	152	230	107	489
Age	76.1	75.0	75.2	75.4
Gender (male)	56.5%	66.1%	50.0%	59.7%
Education (years)	16.3	15.8	14.7	15.7
MMSE at baseline	29.2	27.1	23.5	27.0
MMSE at 24 months	29.1	25.3	18.9	25.2
#ε2ε3	18 (11.8%)	10 (4.3%)	2 (1.9%)	30 (6.1%)
#ε2ε4	2 (1.3%)	7 (3.0%)	2 (1.9%)	11 (2.2%)
#ε3ε3	94 (61.8%)	91 (39.6%)	28 (26.1%)	233 (43.6%)
#ε3ε4	35 (23.0%)	89 (38.7%)	54 (50.1%)	178 (36.4%)
#ε4ε4	3 (2.0%)	33 (14.3%)	21 (19.6%)	57 (11.7%)
ADNI-MEM	0.996	−0.058	−0.889	0.0876
ADNI-EF	0.743	0.0434	−0.905	0.0532
#Significantly declined (MMSE drops >= 3)	2 (1.3%)	77 (33.5%)	60 (56%)	139 (28.4%)

This table shows features and statistics of the ADNI samples used in this study.
