# Supplementary material for: COMPASS: A computational model to predict changes in MMSE scores 24-months after initial assessment of Alzheimer’s disease
Source: Sci Rep. 2016 Oct 5;6:34567. doi: 10.1038/srep34567 (PMC5050516; doi:10.1038/srep34567)
Supplement: Supplementary Info S2 [file srep34567-s2.doc]

**COMPASS: A computational model to predict changes in MMSE scores 24-months after initial assessment of Alzheimer's disease**

Fan Zhu, Bharat Panwar, Hiroko H. Dodge, Hongdong Li, Benjamin Hampstead, Roger L. Albin, Henry L. Paulson and Yuanfang Guan

**Comparison Methods Implemented using WEKA**

The comparison methods used in this paper are all implemented using WEKA (Waikato Environment for Knowledge Analysis). Weka is a collection of machine learning algorithms for data mining tasks developed by the Machine Learning Group at the University of Waikato (<http://www.cs.waikato.ac.nz/ml/index.html>). WEKA 3.6 is used in our experiments.

The build-in cross-validation of WEKA with Pearson correlation coefficient is used to evaluate the performance of all the comparison methods. For Gaussian process regression, linear regression, Radial basis function (RBF) Network, decision tree and support vector machine for regression (SMOReg), default parameters are used. For SMOReg, we have also evaluated the polynomial kernel of WEKA’s SVM function with E = 2 and 3.

*RBF Network sometimes generates results with consistent negative correlation coefficient. Hence we used the absolute value of correlation coefficient values as its performance.
